# Integration of metatranscriptomics data improves the predictive capacity of microbial community metabolic models

**DOI:** 10.1093/ismejo/wraf109

**Published:** 2025-05-31

**Authors:** Yunli Eric Hsieh, Kshitij Tandon, Heroen Verbruggen, Zoran Nikoloski

**Affiliations:** Systems Biology and Mathematical Modeling Group, Max Planck Institute of Molecular Plant Physiology, 14476, Potsdam, Germany; Bioinformatics Department, Institute of Biochemistry and Biology, University of Potsdam, 14476, Potsdam, Germany; School of BioSciences, The University of Melbourne, Parkville, VIC, 3010, Australia; School of BioSciences, The University of Melbourne, Parkville, VIC, 3010, Australia; School of BioSciences, The University of Melbourne, Parkville, VIC, 3010, Australia; CIBIO, Centro de Investigação em Biodiversidade e Recursos Genéticos, InBIO Laboratório Associado, Campus de Vairão, Universidade do Porto, 4485-661, Vairão, Vila do Conde, Portugal; Systems Biology and Mathematical Modeling Group, Max Planck Institute of Molecular Plant Physiology, 14476, Potsdam, Germany; Bioinformatics Department, Institute of Biochemistry and Biology, University of Potsdam, 14476, Potsdam, Germany

**Keywords:** community metabolic modeling, metatranscriptomics, data integration, microbial community, metabolic interaction

## Abstract

Microbial consortia play pivotal roles in nutrient cycling across diverse ecosystems, where the functionality and composition of microbial communities are shaped by metabolic interactions. Despite the critical importance of understanding these interactions, accurately mapping and manipulating microbial interaction networks to achieve specific outcomes remains challenging. Genome-scale metabolic models (GEMs) offer significant promise for predicting microbial metabolic functions from genomic data; however, traditional community GEMs typically rely on species abundance information, which may limit their predictive accuracy due to the absence of condition-specific gene expression or protein abundance data. Here, we introduce the Integration of Metatranscriptomes Into Community GEMs (IMIC) approach, which utilizes metatranscriptomic data to construct context-specific community models for predicting individual growth rates and metabolic interactions. By incorporating metatranscriptomic profiles, which reflect both gene expression activity and partially encode abundance information, IMIC could predict condition-specific flux distributions that enable the investigation of metabolite interactions among community members. Our results show that growth rates predicted by IMIC correlate strongly with relative as well as absolute abundance of species and offer a streamlined, automated procedure for estimating the single intrinsic parameter. Specifically, IMIC results in improved predictions of measured metabolite concentration changes compared with other approaches in our case study. We further demonstrate that this improvement is driven by the network-wide adjustment of flux bounds based on gene expression profiles. In conclusion, the IMIC approach enables the accurate prediction of individual growth rates and improves the model performance of predicting metabolite interactions, facilitating a deeper understanding of metabolic interdependencies within microbial communities.

## Introduction

Microbial communities are essential for maintaining nitrogen, carbon, and sulfur cycles important to all ecosystems [1–4]. The composition and functionality of these communities are profoundly shaped by the interactions between community members facilitated via exchange of metabolites [5]. Recent advancements in metagenomics, coupled with mechanistic metabolic modeling, have enabled the direct functional characterization of naturally occurring microbial communities through the generation and analysis of genome-scale metabolic models (GEMs) [6]. For instance, GEMs have been applied to analyze metabolic interactions within microbial communities and to design synthetic communities aimed at enhancing agricultural productivity [7, 8].

Flux balance analysis (FBA), traditionally employed to model flux distributions and growth of single species, has been extended to computationally model microbial communities [9]. This extension involves using a compartmentalized GEM approach that is analyzed by constraint-based modeling approaches that impose steady-state, thermodynamic, and other biophysical constraints, as seen in approaches and tools such as Microbiome Modeling Toolbox [10], cFBA [11], MICOM [12], and SteadyCom [13]. In the compartmentalized GEM, genes, reactions, and metabolites from various taxa are integrated into a single GEM, with separate compartments designated for each taxon [9]. These compartments are linked via another compartment that represents the extracellular environment [14]. This setup enables the modeling of metabolite exchanges between community members, providing insights into interspecies interactions.

The true strength of constraint-based modeling of microbial communities lies in its ability to predict the growth of the community and its individual members. To this end, the objective function in community GEMs is often the maximization of community growth, calculated as the sum of the individual growths weighted by their relative abundances [10]. We note that this modeling assumption is applicable to both mutualistic communities and communities in which members compete for metabolic resources, whereby direct competition through toxicity effects between community members is not considered. In addition, achieving maximum community growth does not necessarily correspond with optimal growth of each individual species in the community; some species may exhibit high growth rates, while others may not grow at all. One way to address this issue is to employ a two-step optimization strategy, as performed in MICOM [12]: In the first step, the community growth rate, given by the sum of individual growth rates weighted by the abundance of individual microbes, is maximized. As a result, the objective function in the first step enforces that highly abundant microbes will tend to be associated with larger growth rates predicted by the community GEM. In the second step, the sum of squared growth rates of individual microbes in the community is minimized while ensuring that at least a proportion, $\alpha$, of the optimal community growth is achieved; the parameter $\alpha$ is termed cooperative trade-off.

While constraint-based modeling has demonstrated the capability to predict growth rates, most existing GEMs provide a genome-centric view based on known metabolic reactions catalyzed by the enzymes encoded in the genome, but they do not account for actual gene expression or protein abundance [15–17]. Lack of such information can limit the accuracy of models in predicting *bona fide* metabolic interactions under specific conditions. This limitation is particularly prominent in community GEMs, where, despite accurate predictions of community growth, the depicted metabolite interactions may not represent actual interactions. This discrepancy arises because multiple flux distributions can achieve the same objective values, leading to uncertainties in predicting metabolic interactions.

One viable approach to enhance the accuracy of flux estimations within community GEMs is to integrate omics data gathered from the community [16, 18, 19]. For instance, GEMs contain the information of gene–protein reaction (GPR) rules, which enable the integration of gene expression data to determine the active and inactive states of reactions within the metabolic network. Various constraint-based approaches have been developed to integrate transcriptomic data into GEMs [18, 20–22]. These have been categorized into three main families [20], namely, (i) Gene Inactivity Moderated by Metabolism and Expression (GIMME)–like approaches that aim to minimize the use of reactions with low gene expression support while maintaining a specified biological objective [23, 24]; (ii) integrated Metabolic Analysis Toolbox (iMAT)–like approaches that identify flux distributions aligning with groups of reactions classified as active or inactive based on gene expression data [25, 26], and (iii) the Model-Building Algorithm (MBA)–like approaches that retain only core reactions, defined by multiple omics data and robust biochemical evidence, and include the minimum necessary noncore reactions to ensure that all reactions can be active [27–29]. Each of these approaches can potentially be extended to community-level models but faces inherent limitations. For instance, GIMME-like approaches perform FBA-based analysis with a cut-off value for considering a reaction active, which may be challenging to specify in a community context. Further, iMAT-like approaches rely on mixed integer linear programming (MILP) that becomes computationally expensive as the community size increases. Lastly, MBA-like approaches generate context-specific metabolic models without providing flux distributions, which may render it difficult to quantify the metabolite interactions within communities.

Another popular approach to integrate transcriptomic data, the E-flux approach, does not rely on discretizing the transcriptomic data but scales the upper bounds of fluxes according to the expression of the genes in the GPR rules relative to the maximum expression in the analyzed data [30]. An extension to E-flux has shown that it facilitates the integration of time-resolved transcriptomic (and metabolomic) data [31]. A recently proposed framework, CoCo-GEMs, combines MICOM with the E-flux approach to integrate metatranscriptomic data into community models [32]. CoCo-GEMs builds on the two-step optimization of MICOM and relies on integration of microbial abundance data, which include the cooperative trade-off as a parameter. Like E-flux, CoCo-GEMs scales the upper bound of reaction fluxes by introducing two parameters: (i) a scaling factor, $\delta$, that adjusts the transcript count sums for each organism and (ii) a regulating factor, $\gamma$, that influences the impact of gene-set expression changes on the flux bounds of the corresponding reactions [32]. As a result, the performance of CoCo-GEMs depends on these three user-defined parameters, including the cooperative trade-off value from MICOM, a scaling factor, and a regulating factor, which may affect the prediction of flux distributions. Consequently, there are no computational approaches permitting the construction of community GEMs without manual adjustments of user-specific parameters.

Here, we introduce IMIC (Integration of Metatranscriptomes Into Community GEMs), an automated approach that utilizes only metatranscriptomic data to develop context-specific metabolic models for community analysis. Although obtaining high-quality metatranscriptomic data is not easy, such data offer the dual advantage of partially capturing abundance information and reflecting the functional activity of microbial communities. IMIC leverages these features to estimate condition-specific flux distributions that enable the investigation of metabolite interactions among community members. Unlike other approaches, IMIC provides procedures for the automated determination of the single parameter appearing in its formulation. Provided stoichiometric models, generated from reference genomes or metagenome-assembled genomes (MAGs), IMIC can predict growth rates of individual community members based on metatranscriptomic data. It identifies key reactions that drive growth rates within the community’s metabolic network and pinpoints the dependence of metabolic interactions on the environmental context. Compared to traditional computational approaches to community modeling, IMIC improves the capability to predict metabolite interactions by integrating metatranscriptomic data into community GEMs.

## Materials and methods

### Metagenome-assembled genomes and metatranscriptomic data

**Case study I:** We made use of 14 high-quality MAGs, each exhibiting >90% completeness and <5% contamination. These MAGs were paired with metagenome and metatranscriptomic data collected at five different time points during the fermentation of Korean traditional soy sauce (ganjang), as previously described [33]. The experiment aimed to delineate the fermentative processes and microbial features in ganjang fermentation over time. The data collection points included 20, 40, 60, 90, and 180 days, providing a comprehensive timeline of fermentation activity. The data were downloaded from the NCBI BioProject accession number PRJNA613738.

**Case study II:** We used data from a synthetic community with two model microorganisms, *Escherichia coli* K-12 and *Pseudomonas putida* KT2240 [34]. These bacteria were cocultured at varying initial ratios of *E. coli* to *P. putida*: 1:1, 1:1000, and 1000:1. Metatranscriptomic data were collected at multiple points (0, 4, 8, and 24 h, during cultivation) to monitor temporal changes in gene expression. Species-specific quantitative PCR was utilized to quantify bacterial growth. Metatranscriptomic data and complete genomes for *E. coli* and *P. putida* were obtained from NCBI BioProjects PRJNA675662, PRJNA225, and PRJNA267.

### Calculation of metagenome-assembled genome abundance, replication rate, and gene expression

MAG abundances were determined by aligning metagenomic sequencing reads against high-quality MAGs using the BWA-MEM algorithm [35]. This alignment was conducted adhering to the best-match criteria, requiring a minimum identity of 90% and an alignment length of at least 20 bp. The abundances were quantified by the count of reads aligned to each MAG by using CoverM v0.7.0 [36] and were quantified relative to the total numbers of mapped reads.

The replication rate of each MAG at different timepoints was determined using CoPTR v1.1.2 [37]. Briefly, all MAGs were compiled to construct a reference database, which was then utilized for read mapping. Following the mapping, genome-wide coverage was calculated for each reference genome. In the final step, peak-to-trough ratios were estimated from the coverage maps to infer replication rates. To address the compositional heterogeneity inherent to the different timepoints, the minimum number of reads required per MAG was set to 5000, following the default setting, and the minimum number of samples per genome was reduced to one.

The quantification of gene expression was conducted through the alignment of mRNA sequencing reads to MAGs, utilizing the BWA-MEM algorithm [35]. Like for the analysis of MAGs, this process adhered to best match criteria requiring a minimum sequence identity of 99% and an alignment length of no <20 bp. Subsequently, the quantification of read alignments to individual genes within the genomic annotations was performed using the FeatureCounts program [38]. The expression abundance was normalized using transcripts per million (TPM). Additionally, TPM was transformed into a log2 scale to comply with the requirement of CoCo-GEMs approach [32].

### Genome-scale metabolic model and community model reconstruction

Starting from MAGs, the draft genome-scale metabolic models (GEMs) were reconstructed with CarveMe [39], gapseq [40], KBase [41], and the consensus approach [42] following previously described methods [43]. Briefly, reaction and metabolite identifiers from the draft models produced by CarveMe, gapseq, and KBase were standardized to MNXref IDs [44]. For the consensus model construction, the CarveMe model served as the initial template, with other models iteratively merged. During this merging process, model fields were harmonized, and gene identifiers were compared to ensure that any genes absent in the consensus model were added. Reaction comparisons were based on reaction IDs, gene–protein–reaction (GPR) rules, associated metabolites, and mass balance; reactions absent in the consensus model were incorporated accordingly. Duplicate reactions and metabolites were subsequently removed to eliminate redundancies. To obtain biologically meaningful GEMs, gap-filling was performed using COMMIT v1.1.2 [42], employing an M9-anoxic minimal medium for a bacterial community of 14 MAGs and Luria-Bertani (LB) medium for the synthetic community as the growth medium. After gap-filling, individual GEMs were integrated into a unified stoichiometric matrix, assigning each species to a unique compartment, while sharing a common extracellular space for the creation of the community model.

### Integration of metatranscriptomic data into community models

To integrate metatranscriptomic data into a microbial community model allowing the prediction of individual growth rates and metabolite interactions within the community, we developed the IMIC approach. IMIC utilizes GPR rules to transform gene expression data into constraints on reaction fluxes. To prevent essential reactions from being blocked by these constraints, a relaxation parameter ($\beta$) is introduced. The objective of IMIC optimizes the trade-off between the sum of individual growth rates within the community and the sum of relaxations to the metatranscriptomic-derived upper bounds on the fluxes. The objective is formulated as a linear programming (LP) problem as follows:


$$ \mathit{\max}{\sum}_j{\mu}_j-\lambda \sum_{i,j}\left({\beta}_{ij}^{+}+{\beta}_{ij}^{-}\right) $$

*s.t.*


$$ \mathbf{S}\bullet \mathbf{v}=\mathbf{0} $$

$$ 0\le{v}_{i,j}\le{v}_{\mathit{\max},i,j}\times \left(\frac{f_{i,j}\left(\overset{\sim }{g}\right)}{M}+{\beta}_{ij}^{+}-{\beta}_{ij}^{-}\right)\kern0.75em i\in R\ have\ GPR $$

$$ 0\le{v}_{i,j}\le{v}_{\mathit{\max},i,j} $$

$$ 0\le \frac{f_{i,j}\left(\overset{\sim }{g}\right)}{M}+{\beta}_{ij}^{+}-{\beta}_{ij}^{-}\le 1 $$

$$ \mathbf{0}\le{\boldsymbol{\mathrm{\beta}}}^{+},{\boldsymbol{\mathrm{\beta}}}^{-}\le \mathbf{1000} $$

Here, ${\mu}_j$ is the growth rate of MAG $j$, $\lambda$ represents a balancing factor between growth optimization and relaxation, ${\beta}_{ij}^{+}$ and ${\beta}_{ij}^{-}$ are relaxation parameters, ${v}_{i,j}$ denotes the reaction flux of reaction $i$ in the model of MAG $j$, and ${f}_{i,j}\left(\overset{\sim }{g}\right)$ is calculated based on GPR rules as follows:


$$ {f}_{i,j}\left({g}_{i,j,1}\wedge{g}_{i,j,2}\right)=\min \left\{\theta \left({g}_{i,j,1}\right),\theta \left({g}_{i,j,2}\right)\right\} $$

$$ {f}_{i,j}\left({g}_{i,j,1}\vee{g}_{i,j,2}\right)=\mathrm{sum}\left\{\theta \left({g}_{i,j,1}\right),\theta \left({g}_{i,j,2}\right)\right\} $$

where $\theta \left({g}_{i,j,1}\right)$ represents the expression abundance (TPM) of gene ${g}_{i,1}$ in MAG $j$, $M$ is the maximum value of $f\left(\overset{\sim }{g}\right)$ over all MAGs in the community. All the simulations in this study were performed in Matlab 2023b [45] with the Gurobi solver v11.00 [46].

### Determination of the balancing factor $\boldsymbol{\lambda}$

In the IMIC framework, we introduced a user-specified factor, $\lambda$, that balances the sum of growth rates and the relaxation parameter. The determination of $\lambda$ can follow two approaches: (i) For users having abundance data for each organism within the community, $\lambda$ can be determined through a cross-validation approach. Specifically, the dataset is randomly partitioned into $k$ subsets. Each subset sequentially acts as the test group with the remainder serving as the training set. This process evaluates different $\lambda$ values, ranging from (0.1, 0.5, 1, 2, 3, ... 50), to ensure a comprehensive representation of the dataset. The correlation between the predicted individual growth rates and the relative abundance of each organism is analyzed. The optimal value of $\lambda$ is then determined using the average of $\lambda$ values that yield the best performance across multiple trials. (ii) In the absence of abundance data, sensitivity analysis can be employed by testing $\lambda$ values ranging from (0.1, 0.5, 1, 2, 3, ... 50) to calculate the corresponding values for the objective of the linear programming problem mentioned above. The optimal value of $\lambda$ corresponds to the transition (i.e. inflection) point of the curve, where the growth and relaxation become balanced.

### Assessing the precision of growth rates predicted by IMIC

To evaluate the precision of predicted growth rates within the community, a growth rate variability analysis was conducted for each organism using the following LP problem:


$$ \mathit{\max}/\mathit{\min}{\mu}_j $$

*s.t.*


$$ \mathbf{S}\bullet \mathbf{v}=\mathbf{0} $$

$$ 0\le{v}_{i,j}\le{v}_{\mathit{\max},i,j}\times \left(\frac{f_{i,j}\left(\overset{\sim }{g}\right)}{M}+{\beta}_{ij}^{+}-{\beta}_{ij}^{-}\right)\kern0.75em i\in R\ have\ GPR $$

$$ 0\le{v}_{i,j}\le{v}_{\mathit{\max},i,j} $$

$$ 0\le \frac{f_{i,j}\left(\overset{\sim }{g}\right)}{M}+{\beta}_{ij}^{+}-{\beta}_{ij}^{-}\le 1 $$

$$ \mathbf{0}\le{\boldsymbol{\beta}}^{+},{\boldsymbol{\beta}}^{-}\le \mathbf{1000} $$

$$ \sum_j{\mu}_j-\lambda \sum_{i,j}\left({\beta}_{ij}^{+}+{\beta}_{ij}^{-}\right)={z}^{\ast } $$

Here, the objective function value from the IMIC approach, ${z}^{\ast }$, was computed and used as a constraint to determine the maximum and minimum predicted growth rates for each organism within the community with the fixed balancing parameter $\lambda$.

### Identification of key reactions within a community

The common reactions across the community of 14 bacterial MAGs were identified, excluding those lacking GPR associations. The flux values for the remaining common reactions were calculated using the optimal value of $\lambda$, calculated based on the proposed automated procedure. To assess the relationship between the flux of each common reaction and the relative abundance of each MAG, Spearman correlation analysis was conducted. The *P*-values were corrected for multiple hypothesis testing using the Benjamini–Hochberg procedure [47]. Reactions with a Spearman correlation coefficient >0.7 and corrected *P* -value <.05 were considered as key reactions, hypothesized to drive the growth rates of individual organisms within the community. Further analysis was conducted to elucidate the biological relevance of these key reactions. We examined the correlation between the flux of each key reaction and its corresponding correction factor $ f\left(\overset{\sim }{g}\right) $ in each MAG. Additionally, the impact of these key reactions on community growth was assessed by systematically knocking out each key reaction and reevaluating the community model using the IMIC framework with the optimal $\lambda$ value.

### Quantification of interactions within the community

We focused on the common import reactions, transporting the metabolites from the extracellular environment into the intracellular space of a community model, among 14 MAGs, and conducted a minimum flux sum analysis [48] for these transported metabolites. This analysis, using both the MICOM and IMIC frameworks, aimed to evaluate the reliability of metabolite interactions revealed by the IMIC methodology. We further evaluated model predictions of concentration changes for 4 sugars (i.e. mannose, glucose, galactose, and fructose), 2 organic acids (i.e. acetate and lactate), and 11 amino acids (i.e. alanine, arginine, asparagine, aspartate, glutamate, glycine, leucine, lysine, proline, threonine, and valine) in the extracellular space. The metabolite concentrations were estimated using flux sum analysis [48] by solving the optimal objective function across IMIC, MICOM, and CoCo-GEM, under conditions with and without the integration of abundance data. To validate model performance, the predicted concentrations of the 11 amino acids were compared with measured concentrations during fermentation [49]. Measurement data were extracted from the bar plot in Fig. 5 of Han *et al*. [49] using the WebPlotDigitizer tool [50].

In a second case study, we examined the interaction between *E. coli* and *P. putida* within a synthetic two-bacterial community model using the IMIC approach, following the minimum flux sum analysis mentioned above but extending the analysis to encompass all metabolites present in the extracellular space. To further characterize specific metabolite interactions influenced by different initial coculture ratios, we selected the top 20 metabolites based on their minimum flux sum values. This comparison highlighted key metabolites taken up from the environment under varying initial coculture ratios, thereby elucidating specific metabolic dependencies shaped by the initial population structures.

## Results

### IMIC provides fully automated integration of metatranscriptomics data into microbial community models

**Figure 1:**
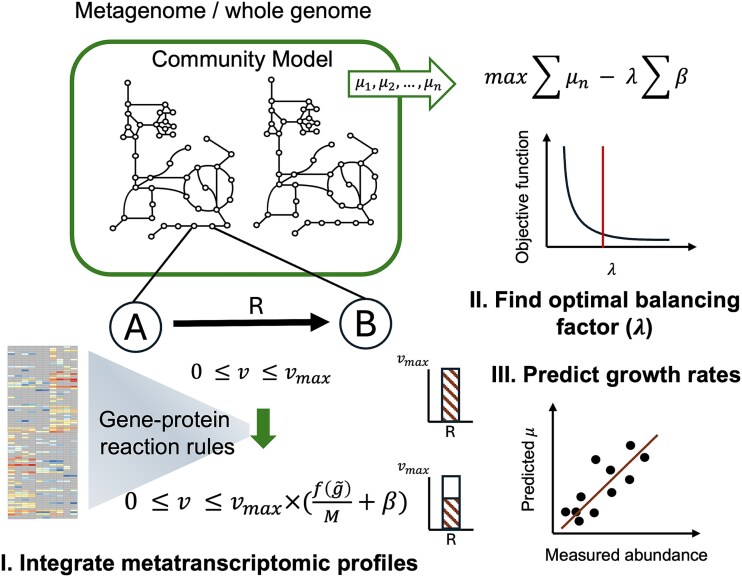
Illustration of the workflow underlying the IMIC approach. IMIC employs metatranscriptomic data to constrain reaction fluxes, $v$, in accordance with GPR rules, utilizing a data-derived rescaling factor $f\left(\overset{\sim }{g}\right)/M$ for the maximum flux, ${v}_{max}$, and relaxation parameters, $\boldsymbol{\mathrm{\beta}}$, that ensure simulation of growth. The objective function of IMIC balances the trade-off between maximizing the sum of growth rates, ${\mu}_n$, of community members and minimizing the aggregate of the relaxations. The parameter $\lambda$ represents a balancing factor between growth optimization and relaxation and is determined by sensitivity analysis or cross-validation. The determined value of $\lambda$ is then used to obtain predictions of growth rates for all members in the community using the objective.

Here, we first asked if growth rates of individual microbes in a community can be predicted without directly using microbial abundance data. To address this question, we devised a constraint-based modeling approach, which we term IMIC (Fig. 1, Materials and Methods). As a flux-balance-based approach [51], IMIC assumes that the community operates at steady state while maximizing an objective function. Specifically, IMIC involves the following: (i) constraining reaction fluxes using metatranscriptomic data, which is achieved through the GPR rules. To this end, a correction factor, $f\left(\overset{\sim }{g}\right)$, is first calculated for the maximum flux for each reaction. This factor is then scaled by the maximum value of $f\left(\overset{\sim }{g}\right)$ across all reactions in the community GEM. Conceptually, this step of IMIC is similar to the E-flux approach and its extensions [30, 31, 52]. However, metatranscriptomic data are often sparse, with relatively few genes showing considerable expression, which can be influenced by taxon and/or gene–family abundance [53–55]. As a result, we introduced a reaction-specific relaxation parameter, ${\beta}_i$, for a reaction $i$ whose flux is bound by metatranscriptomic data. This ensures that the sparsity of metatranscriptomic data does not lead to blocking of essential reactions, which could significantly impact growth predictions. With these constraints, IMIC maximizes the sum of individual growth rates while minimizing the sum of relaxation parameters, controlled by a balancing factor, $\lambda$. (ii) Determining the value of the balancing factor$, \lambda$, which is used in prediction of abundances. Clearly, varying the value of $\lambda$ is expected to result in different predictions of growth for the individual microbes in the community GEM. To determine the optimal value of $\lambda$, that best aligns with measurement of relative abundances, IMIC does not rely on abundance data. Instead, the optimal value for $\lambda$ is identified by conducting sensitivity analysis for the objective function of IMIC (see Materials and Methods). (iii) Predicting individual growth rates by leveraging the selected value for the balancing factor, $\lambda$, constraints from metatranscriptomic data, and relaxations. As a result, IMIC provides a framework for understanding the contribution of microbial metabolic pathways and interactions to growth dynamics.

**Figure 3:**
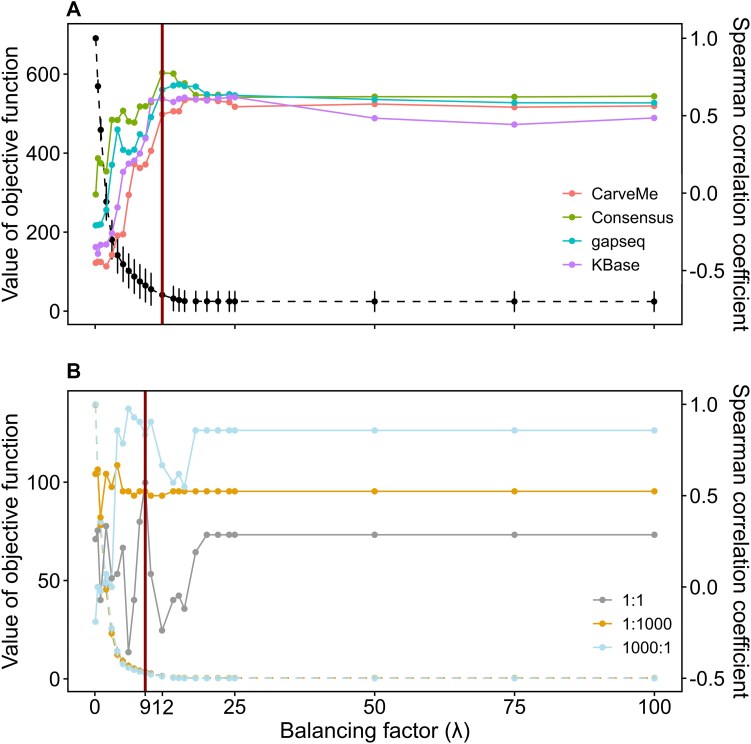
Effects of the balancing factor on the performance of IMIC. The performance of IMIC was evaluated over different values for the balancing parameter, $\lambda$. (A) presents the outcomes for a bacterial community involved in ganjang fermentation, where solid lines denote the Spearman correlation coefficient between the predicted growth rates and the measured MAGs’ relative abundance. Different colors correspond to community models derived from different reconstruction approaches, while the dashed line depicts the sensitivity analysis of the consensus community model. (B) illustrates the results for a two-species synthetic community comprising *E. coli* and *P. putida*. Here, solid lines indicate the Spearman correlation coefficient between the predicted growth rates and cell quantities, with colors distinguishing initial species ratios of 1:1, 1000:1, and 1:1000. Dashed lines denote the outcomes from the sensitivity analysis for the community model given varying initial ratios. Red lines in both (A) and (B) highlight the transition (i.e. inflection) points in curves resulting from the sensitivity analysis. The transition points are used to identify the optimal value of the balancing factor, $\lambda$.

### Comparative analysis of the predictive performance of IMIC and its contenders

In the comparative analysis, we examined the performance of both MICOM [12] and CoCo-GEMs [32] against that of IMIC, by using metagenome and metatranscriptomics data collected at the different time points to investigate the fermentation features of ganjang [33]. The bacterial community is described by 14 high-quality MAGs capturing the temporal dynamics of fermentation (see Materials and Methods). An average of 84.48% of metagenomic reads and 80.41% of metatranscriptomic reads were successfully mapped to the 14 representative MAGs. These results confirm that the selected MAGs sufficiently captured the dominant members of the microbial community and were representative of the underlying genomic and transcriptomic profiles. The comparative analysis considered two scenarios—with and without direct integration of abundance data—using GEMs reconstructed from the consensus approach [42]. To determine the optimal cooperative trade-off parameter in MICOM for models reconstructed using CarveMe [39], gapseq [40], KBase [41], and consensus models [42], we compared model performance with abundance data using trade-off values of 0.3, 0.5, 0.7, 0.9, and 1. The consensus model achieved the best performance with a trade-off value of 0.3, whereas a value of 0.5 was optimal for CarveMe, gapseq, and KBase models. These selected values were also used in the scenario without abundance data.

**Figure 4:**
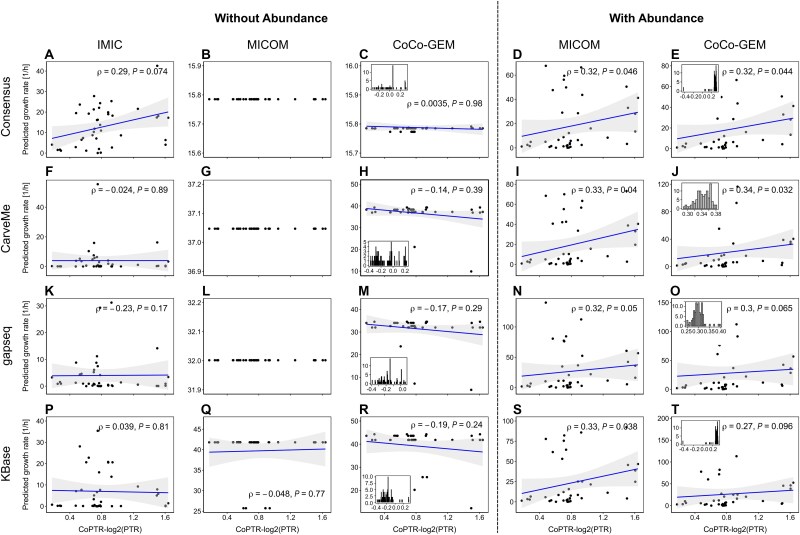
Comparative analysis of model performance using replication rates. IMIC, MICOM, and CoCo-GEM frameworks were applied to bacterial community models of 14 MAGs reconstructed using the consensus [1], CarveMe [2], gapseq [3], and KBase [4] approaches. Model performance was evaluated by comparing predicted growth rates with replication rates using Spearman correlation. For IMIC, a balancing factor (λ) of 12 was utilized. MICOM and CoCo-GEMs were assessed both with and without the integration of abundance data. The performance of CoCo-GEMs was further examined across varying parameter values (γ and δ), ranging from 10 to 100 in increments of 10. The panels for CoCo-GEM display the distribution of Spearman correlation coefficients corresponding to the various parameter combinations. Scatter plots within the CoCo-GEM panels present data points where correlation coefficients are equal to or approximate the mean Spearman correlation value for the respective parameter configurations. The shaded area in all panels indicates the 95% confidence interval.

**Figure 2:**
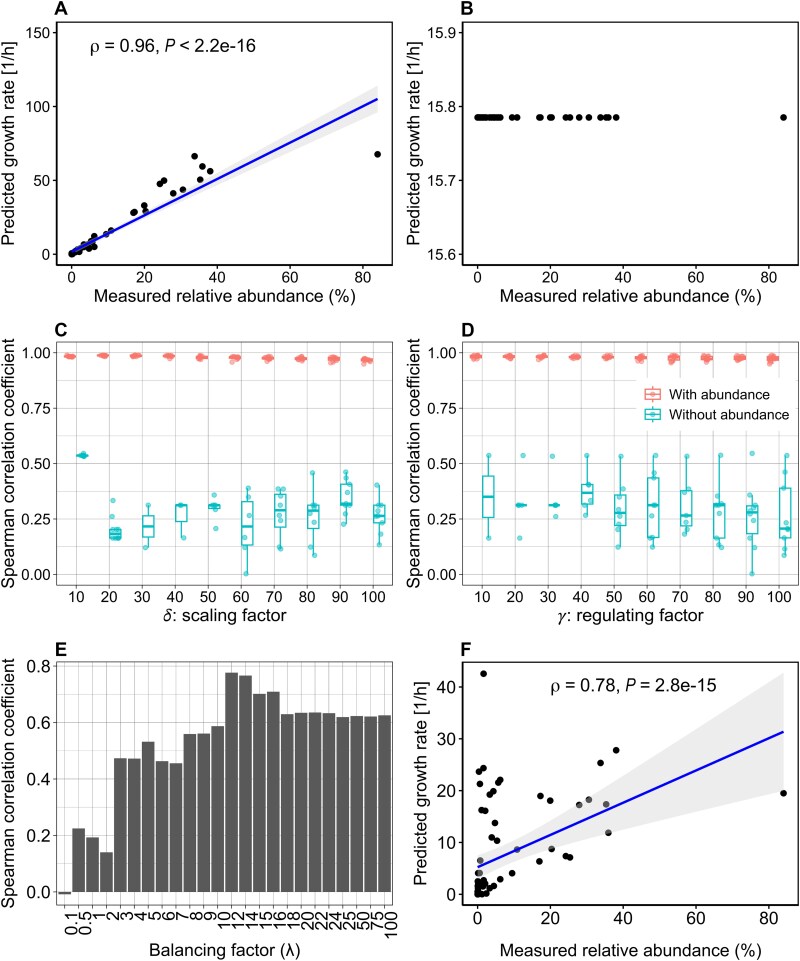
Performance of MICOM, CoCo-GEMs, and IMIC in predicting individual growth rates. The consensus community model, comprising 14 bacterial species, was employed in the MICOM, CoCo-GEMs, and IMIC frameworks separately to predict individual bacterial growth rates. Spearman correlation analysis between the predicted individual growth rates and the relative abundance of each species was conducted. In both MICOM and CoCo-GEMs, the frameworks were tested with and without integration of abundance data. The performance of CoCo-GEMs was investigated for different values of the parameters $\delta$and $\gamma$, ranging from 10 to 100 with increments of 10. (A) depicts growth rates within the microbial community as predicted by MICOM with a cooperative trade-off value of 0.3, utilizing individual abundance data to adjust the predictions. (B) illustrates MICOM’s performance when abundance data are not employed, with all abundances set to a uniform value of one for all bacterial species in the community model. (C) displays how variations in parameter $\delta$ influence the correlation coefficients in CoCo-GEMs. (D) depicts the impact of variations in parameter $\gamma$ on the Spearman correlation coefficients. The performance of IMIC is shown in (E) for different values for the balancing factor of $\lambda$. (F) shows the optimal performance of IMIC by using a balancing factor of 12.

We found that MICOM demonstrated limited capability in predicting individual growth rates without abundance data, given that all species within the community were assigned identical growth rates (Fig. 2A and B). We further examined the influence of the two additional parameters, $\delta$ and $\gamma$, used in CoCo-GEMs. For these evaluations, the cooperative trade-off values applied to models reconstructed using different approaches were consistent with those used in MICOM. We found that the inclusion of abundance data resulted in predictions of individual growth rates closely matching the measured relative abundances, regardless of parameter settings (Fig. 2C and D). However, when abundance data were not considered, achieved by setting a uniform value of one for all bacteria in the community, the ability to accurately predict individual growth rates varied significantly depending on the specific values of $\delta$ and $\gamma$ applied. The results indicated that, for most tested parameter combinations, the Spearman correlation was on average smaller than 0.5. These results underscored the strong dependence of MICOM and CoCo-GEMs on abundance data to accurately predict individual growth rates in the community. In comparison, with the same community model, IMIC resulted in the best Spearman correlation coefficient of 0.78 for the value of the balancing parameter lambda of 12 (Fig. 2E and F).

To further examine the effect of the models used on the performance of IMIC, we considered bacterial community models reconstructed using three approaches, namely, CarveMe [39], gapseq [40], and KBase [41], used in the generation of the consensus models [42], discussed above (Fig. 3A). We found that the integration of metatranscriptome data in the consensus community model using IMIC generally outperformed the models obtained from the other approaches (Supplementary Fig. S2). The improved predictive performance of integrating metatranscriptomics data into consensus community models is likely due to the more comprehensive gene set included in such models [43], allowing for constraining more reaction fluxes with the available metatranscriptome data.

**Figure 5:**
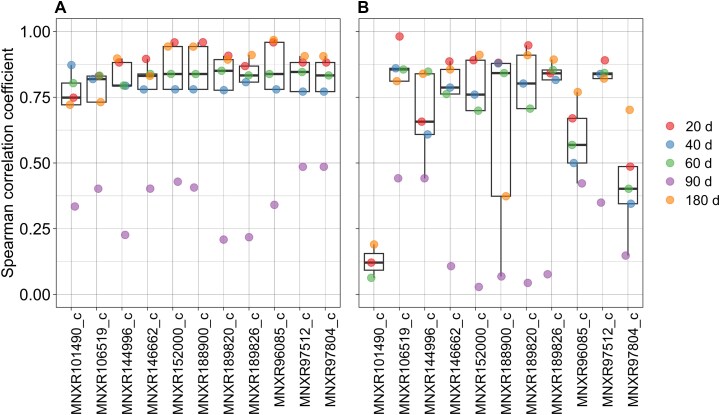
Correlation analysis of key reaction fluxes with relative abundance of MAGs and correction factor ($\boldsymbol{f}\left(\overset{\sim }{\boldsymbol{g}}\right)$). The key reactions in the community of 14 bacterial MAGs were identified by comparing the reaction flux and the relative abundance of each MAG. (A) presents the Spearman correlation analysis between the flux values of key reactions and the relative abundance of MAGs at different time points. (B) displays the correlation between the flux values of key reactions and the flux correction factor $f\left(\overset{\sim }{g}\right)$), which were derived from gene expression values, at different time points. Each time points are represented in a different color, indicated in the legend. The reactions corresponding to the names shown on the *x*-axes are detailed in Supplementary Table S1.

We further conducted a comprehensive comparison of predicted growth rates with replication rates, as similar analyses were previously performed in studies of MICOM and CoCo-GEM. In this study, we expanded the scope of the comparison by evaluating model performance across different reconstruction approaches using IMIC, MICOM, and CoCo-GEM (Fig. 4). Both MICOM and CoCo-GEM were applied to models derived from various reconstruction methods under scenarios that included and excluded abundance data. For CoCo-GEM, we assessed model performance across a range of parameter values ($\delta$ and $\gamma$), varying from 10 to 100 in increments of 10. Consistent with the findings when comparing to relative abundance, the results revealed that the performance of CoCo-GEM is highly sensitive to the selection of parameter values. Under the scenario without the usage of abundance data, we found that the consensus model consistently outperformed the others, irrespective of whether IMIC or CoCo-GEM was used. In contrast, when abundance data were included in MICOM and CoCo-GEM, all models performed similarly, reflecting the strong constraints imposed by abundance data. Among the frameworks applied to the consensus model, IMIC demonstrated superior performance compared to MICOM and CoCo-GEM in scenarios excluding abundance data. Even when compared to MICOM and CoCo-GEM with abundance data, IMIC achieved comparable performance. Although CoCo-GEM exhibited superior performance under specific parameter settings in the absence of abundance data, the identification of the optimal parameter set required information on replication rates. This additional step introduces redundancy in the use of metabolic models for predicting individual bacterial growth rates in a community, underscoring the streamlined approach of IMIC.

**Figure 6:**
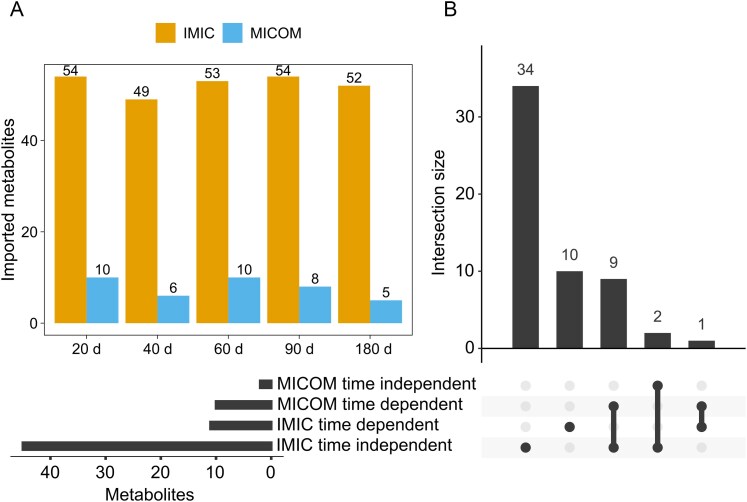
Comparison of imported metabolites between IMIC and MICOM. Essential imported metabolites, derived from common import reactions among 14 MAGs, were identified based on a minimum flux sum value with a threshold of 10^−5^. Metabolites were classified as time-dependent if they were utilized only at specific time points, and as time-independent if they were consistently used across all time points. (A) illustrates the count of imported metabolites at various time points comparing IMIC and MICOM. (B) displays the intersection of metabolites between time-dependent and time-independent categories across both IMIC and MICOM.

Overall, our results suggest that the proposed sensitivity analysis is an effective tool for identifying the optimal $\lambda$ value. In support of this claim, we observed that the sensitivity analysis resulted in a value of 12 for the balancing factor $\lambda$, irrespective of the reconstruction approach used to obtain the community GEM (Fig. 3A). This value was associated with a higher Spearman correlation coefficient (*ρ* = 0.64 on average) between the predicted growth rates and measured relative abundance for all community GEMs, indicating that the optimal value for the single parameter of IMIC can be effectively determined even in absence of abundance data. We also note that similar findings were obtained with another microbial community, including two microbes with different initial coculture ratios (see Materials and Methods), where the sensitivity analysis resulted in a value for the balancing parameter that also corresponded to a higher Spearman correlation coefficient (Fig. 3B).

### IMIC results in precise predictions of growth rates in bacterial community models

Here, we investigated the precision with which IMIC predicts individual growth rates within a bacterial community using variability analysis performed for the growth rate of each MAG in the ganjang community (see Materials and Methods). The relative variability of growth rates for each bacterium within the community was quantified using the ratio of the range to the maximum predicted growth rate; thus, a relative variability value close to zero indicates a more precise prediction of growth rates. This analysis was conducted with community GEMs reconstructed using four different approaches, applying a consistent balancing factor of 12 derived from sensitivity analysis (Supplementary Fig. S3). Additionally, we also evaluated the models with their respective optimal balancing factors [12, 15, 20, 25] identified from model performance (Fig. 3) for the consensus, CarveMe, gapseq, and KBase approaches, respectively (Supplementary Fig. S4). Our results indicated that the consensus model demonstrated superior precision in predicting individual growth rates, regardless of the balancing factor used. This outcome underscores the effectiveness of the IMIC with consensus models in reliably forecasting growth rates within complex bacterial communities.

### IMIC identifies reactions driving growth rates of microbial community members

To determine the key reactions influencing individual growth rates within a bacterial community, we analyzed the Spearman correlation between the flux values of common reactions across all MAGs and the corresponding relative abundances. For the ganjang community, we identified 11 reactions that exhibited statistically significant correlations (Spearman correlation coefficient > 0.7, *P* < .05) with relative abundance at most time points, except for the time point of 90 days (Fig. 5A). Additionally, these reactions showed an association, *ρ* > 0.6 on average, with the flux correction factor $f\left(\overset{\sim }{g}\right)$, calculated based on gene expression values (Fig. 5B). We found that most of these key reactions are purine metabolism, peptidoglycan and lysine biosynthesis, and pentose phosphate pathway (Supplementary Fig. S5 and Supplementary Table S1). To explore the causal impact of these reactions on growth, we conducted individual knockouts of these reactions from the community model using IMIC with a balancing factor $\lambda$ of 12. The impact on community growth rate was assessed, revealing that over half of these key reactions significantly influenced community growth rates, with six reactions potentially blocking community growth when individually knocked out (Supplementary Fig. S6). Consequently, our findings demonstrated that IMIC can be used to effectively identify the drivers of growth rates within the community.

### IMIC enhances the reliability of predictive metabolite interactions in the community

We initially assessed the potential of integrating metatranscriptomic data to enhance the predictive accuracy and reliability of metabolite interactions within a bacterial community. For this purpose, we utilized the IMIC and MICOM frameworks to perform minimum flux sum analysis on each metabolite, transported by common import reactions among the models of the 14 MAGs, aiming to evaluate the capability of each framework in capturing metabolite interactions. The minimum flux sum value with a threshold of 10^−5^ was utilized to determine the imported metabolites essential for optimal community growth and at the optimal value for the balancing parameter.

Within the ganjang community model, we identified 84 common imported metabolites across the 14 MAGs. The IMIC approach revealed an average of 52 essential metabolites used under optimal community growth conditions, whereas MICOM identified an average of only eight such metabolites (Fig. 6A). Further analysis categorized essential imported metabolites into time-dependent and time-independent groups based on their usage at different time points. Most metabolites were found to be time-independent in IMIC (*n* = 45), indicating that they are required for growth over the entire investigated time range (Fig. 6B). In contrast, MICOM identified only two time-independent metabolites. Further, we identified most of time-dependent metabolites in MICOM were time-independent in IMIC. The only metabolite consistently classified as time-dependent in both IMIC and MICOM was L-arginine. These differences suggest that the integration of metatranscriptomic data influences flux distribution, translating into observed differences in predicted metabolic interactions. Overall, IMIC was able to encompass all the essential imported metabolites identified in MICOM, suggesting that IMIC has the ability to elucidate metabolite interactions at a finer detail compared to MICOM.

In previous studies, the concentration changes of free sugars, including mannose, glucose, galactose, and fructose, as well as several amino acids produced during the fermentation process, were monitored across different time points [33, 49]. To compare model prediction results with these experimental measurements, we conducted flux sum analyses for 4 sugars, 2 organic acids, and 11 amino acids existing in the extracellular space in community model, which had been previously quantified, while solving the optimal objective function of IMIC, MICOM, and CoCo-GEM at each time point (Supplementary Fig. S7). Flux sums have been used as proxies for metabolite concentrations because larger fluxes around a metabolite are expected to be driven by larger pools of substrates. For MICOM and CoCo-GEM, the flux sum changes of these targeted metabolites were analyzed under scenarios both with and without the integration of abundance data to evaluate the impact of abundance constraint on the models.

To enable a direct comparison with the findings from a previous study [49], the flux sum value of glucose, galactose, mannose, and fructose was summed and represented collectively as “free sugars” to track the total flux sum changes across time points. Additionally, we performed a quantitative analysis by calculating the correlation between the predicted minimum flux sums, serving as proxies for concentrations, of 11 amino acids and their measured concentrations during the fermentation process (Supplementary Tables S2 and S3). The results from IMIC demonstrated a better alignment between the predicted flux sum values and the experimentally observed concentration changes of metabolites across time points compared to MICOM and CoCo-GEM [49]. Specifically, although the correlations were not statistically significant after multiple hypothesis testing, 9 out of 11 amino acids exhibited a positive correlation (both Spearman and Pearson) between predicted and measured concentrations. Among them, five metabolites, alanine, glycine, leucine, lysine, and valine, showed a high correlation coefficient (>0.7), suggesting a strong positive relationship. In contrast, MICOM failed to reflect these observed changes under the scenario without abundance data, as the flux sum values for all targeted metabolites remained unchanged. When abundance data were incorporated, 6 out of 11 amino acids exhibited positive correlations; however, only two of these correlations were higher than 0.7. For CoCo-GEM, we found that the results were unaffected by the inclusion of abundance data, indicating that the abundance constraint on growth rate and exchange reactions in CoCo-GEM did not influence the flux sum values. Furthermore, CoCo-GEM predictions identified only four metabolites with positive correlations, with only three metabolites, leucine, proline, and threonine, exhibiting a high correlation (>0.7) in the Spearman analysis. These findings highlight the improvement provided by IMIC to reflect metabolite interaction dynamics during the fermentation process.

### IMIC predicts the dynamics of growth and dissects the metabolic interactions in a synthetic community of two bacteria

To demonstrate the unique capabilities of IMIC, we utilized a synthetic community comprising two bacterial species: *E. coli* K-12 and *P. putida* KT2240 [34] (see Materials and Methods). These bacteria were cocultured at varying ratios (*E. coli* to *P. putida*: 1:1, 1:1000, and 1000:1), and a series of metatranscriptomic data (0, 4, 8, and 24 h) were sampled to investigate the molecular interactions within this community. Utilizing the consensus community models of two bacteria [42], we first determined the optimal $\lambda$ value through sensitivity analysis (Fig. 3B). Subsequent comparisons of the IMIC predicted growth rates with experimentally measured cell quantities demonstrated a moderate positive Spearman correlation for both *E. coli* ($\rho$ = 0.64) and *P. putida* ($\rho$ = 0.5). These results underscore the predictions of individual growth rate from IMIC align closely with measurements of bacterial absolute abundance without direct integration of the latter (Supplementary Fig. S8).

Additionally, we investigated the impact of different initial coculturing ratios on metabolite interactions between *E. coli* and *P. putida*. To this end, we identified metabolites within the community that are necessarily exchanged as those with a minimum flux sum larger than 10^−5^. We found that only approximately one-third of metabolites present in the extracellular space (*n* = 299) were necessarily exchanged (Supplementary Fig. S9). Focusing on the top 30 exchanged metabolites, with the largest min flux-sum values, our findings indicated dynamic shifts in metabolite interactions influenced by both culture time progression and variations in initial coculture ratios (Supplementary Fig. S10). Specifically, at an initial ratio of 1000:1 (*E. coli* to *P. putida*), the community exhibited increased uptake of O_2_, L-malate, and NH_4_(+) at the 24-h time point compared to other time points. In contrast, at an initial ratio of 1:1000, these metabolites showed higher uptake during the initial three time points compared to the later time points. Additionally, the minimum flux sum values of H_2_S and sulfate increased over time at an initial ratio of 1000:1, indicating a growing demand for sulfur over time. A similar pattern was observed for Co(2+) at an initial ratio of 1:1000. These results demonstrated the capability of the IMIC to reveal distinct metabolite interactions within microbial communities that provide directly testable hypotheses.

## Discussion

The integration of omics data into GEMs helps in estimating flux distributions under specific conditions. Although numerous tools have been developed for constructing context-specific metabolic models, an efficient method for incorporating omics data into community GEMs without relying on user-defined parameters is lacking. In this study, we evaluated the commonly used community GEMs framework, MICOM [12], along with its extension, CoCo-GEMs [32], both with and without the use of species abundance data. We found that the accurate prediction of individual growth rates within the community by CoCo-GEMs was highly contingent upon the availability of abundance data (Fig. 2A–D). In addition, CoCo-GEMs requires manual adjustments of user-specific parameters. To overcome these limitations and improve the model performance, we developed IMIC, an automated approach that eliminates the need for user-specific parameter specification and integrates metatranscriptomic data constraints into community GEMs, thereby bypassing the direct reliance on individual abundance data. Although we acknowledge that acquiring high-quality metatranscriptomic data from complex communities may be more challenging and expensive than estimating relative abundances, our results demonstrate that metatranscriptomic data provide deeper insights into metabolic interactions within microbial communities—that are otherwise difficult to obtain using abundance data alone.

Similar to existing approaches derived from E-flux, our approach involves constraining reaction fluxes based on gene expression abundance. However, to address the sparsity of metatranscriptomic data, we incorporate relaxation parameters into the IMIC framework. Unlike MICOM, IMIC does not rely on species abundance data to bound exchange reactions, which enhances precision in depicting metabolite interactions within the community. Instead, IMIC utilizes transcriptional activity to represent the functional potential of the microbial community, allowing for a more biologically informed estimation of metabolic interactions. To further investigate the relationship between gene expression levels and microbial abundance, we analyzed the correlation between the expression abundance of genes included in the models and the relative abundance of the corresponding taxa. Spearman correlation was chosen due to the sparsity of the expression data, with ~30% of genes across all taxa containing zero values. The results revealed a correlation coefficient of 0.69 between TPM values and relative abundance, suggesting that abundance information is partially reflected in the transcriptomic profile. To test the impact of this embedded abundance signal on IMIC performance, we removed flux correction factors, $f\left(\overset{\sim }{g}\right)$, whose values were strongly correlated with abundance (Pearson correlation coefficient > 0.7), and repeated the growth prediction analysis. In total, 8.3% of the flux correction factors (over 4000 reactions) were modified. The resulting correlation between predicted growth rates and replication rates was 0.26, compared to 0.29 in the original results, indicating that model predictions are not driven by one-to-one correspondence between gene expression and growth, since the metatranscriptomic data are used to merely constrain the upper bounds of reactions (while accounting for GPR rules). As a result, model performance can be attributed to a network-wide adjustment of flux bounds, improving the resolution of predicted community-level metabolic interactions.

In the IMIC framework, the only user-specified parameter is the balancing factor, $\lambda$. To mitigate bias and reduce computational effort during the selection of an optimal $\lambda$ value, we recommend conducting a sensitivity analysis. This method has proven effective across various community models, regardless of the reconstruction approach or initial coculture ratios, consistently identifying a relatively optimal $\lambda$ value (Fig. 3). For example, in community models reconstructed using gapseq [40] and CarveMe [39], the optimal $\lambda$ value was identified as 20 based on the model performance. However, Spearman correlation analyses revealed only minor differences when the$\lambda$ value was adjusted to 12, as determined through sensitivity analysis. A similar observation was made for the *E. coli* to *P. putida* ratio of 1:1000, where $\lambda$ values from the sensitivity analysis, though not yielding the absolute best model performance, still provided good results. This underscores the practicality and effectiveness of sensitivity analysis in setting the $\lambda$ parameter within the IMIC framework.

In evaluating model performance with and without metatranscriptomic data, we observed inherent limitations in relying solely on the structure of genome-scale metabolic models to elucidate metabolite interactions within microbial communities. Our findings indicate that flux distribution predictions based solely on achieving accurate growth rate predictions at the community scale are unreliable, due to the multitude of solutions that satisfy the same objective function. For example, minimum flux sum analysis identified only 12 essential metabolites out of 84 imported metabolites within the community. In contrast, integration of metatranscriptomic data led to the identification of 56 essential metabolites, highlighting its utility in refining the solution space by imposing specific constraints on flux distributions. Furthermore, we have shown the improvement provided by IMIC in capturing the dynamic changes in metabolite usage across different stages of the fermentation process. This demonstrates the enhanced resolution that metatranscriptomic data provide in defining critical metabolic interactions within community models.

Although metatranscriptomic analysis provides a general overview of community functionality and identifies highly expressed genes [56–59], IMIC goes a step further by illustrating the dynamics of metabolite usage under various conditions. Specifically, IMIC has revealed that the preference for metabolite uptake from the medium changes with different initial coculture ratios and time points. The predictions made by IMIC about metabolic interactions can be used to posit hypotheses that can be empirically tested by conducting metabolomic analyses of the culturing medium. Altogether, IMIC enhances our understanding of interspecies interactions by utilizing metatranscriptome data to uncover interactions that are not directly observable from the data alone.

In this study, we evaluated model performance under the assumption of a direct relationship between growth rates and relative abundances, which is generally valid in microbial systems where growth is the primary determinant of community composition, such as in the early stages of batch culture [60]. However, this assumption may not hold in environments where additional factors, such as dormancy, cell death, and resource limitations, significantly influence microbial populations, as observed in soil ecosystems [61, 62]. Although the systems analyzed in this study align with the growth-dominant assumption, IMIC inherently accounts for transcriptionally active organisms, allowing it to capture microbial activity dynamics. Additionally, the approach can be readily extended to incorporate other layers of biological information, such as metaproteomic data or stable isotope probing, to further refine metabolic predictions in diverse microbial ecosystems.

We found that the model performance was influenced by the initial mixing ratio of community members (Fig. 3B). A plausible explanation is that a dominant species in the community may exhibit synergistic interactions [63], while an equal 1:1 ratio may lead to a balance between inhibitory and activating effects. The inhibitory effects, in particular, cannot be explicitly captured in the current modeling framework, which is applicable to mutualistic communities or communities whose members compete for metabolic resources, but could be addressed by incorporating interaction-specific constraints or dynamic modeling approaches.

We observed cases where predicted growth rates were disproportionately high despite low relative abundance values for certain taxa (Supplementary Fig. S2). Unlike MICOM and CoCo-GEM, which rely on abundance constraints, IMIC predicts growth rates based on flux through the biomass reaction, with reaction constraints derived from metatranscriptomic data when GPR rules are available. This suggests that metabolic activity inferred from transcriptomic constraints does not always directly translate into proportional biomass accumulation, potentially leading to discrepancies where growth rate predictions exceed observed relative abundances. The accuracy of model predictions is also inherently influenced by the quality of MAGs. Although the MAGs used in this study exhibit >90% completeness, residual genome incompleteness may impact metabolic pathway reconstruction, contributing to deviations between predicted and observed growth patterns. Furthermore, gaps in the current knowledge of microbial metabolism and incomplete annotation of gene functions introduce additional limitations, potentially restricting the model’s ability to fully capture microbial metabolic interactions.

In conclusion, the IMIC approach substantially improves the predictive capacity for microbial community interactions and individual species growth rates based on metatranscriptomic data. This study emphasizes the critical role of integrating metatranscriptomic data in refining community metabolic predictions, particularly by narrowing the solution space for flux distributions and improving the depiction of metabolite exchanges within the community. Additionally, the approach offers an automated method for determining the balancing factor ($\lambda$), making it adaptable to various community structures without requiring extensive parameter tuning. It opens the door for further exploration of microbial interactions in diverse environments, including applications in biotechnology, environmental sciences, and synthetic biology. The results of this study provide a foundation for future research aimed at refining community metabolic models and incorporating additional omics data, such as proteomics and metabolomics, to achieve even higher resolution and predictive power.

## Supplementary Material

Supplementary_Files_wraf109

Supplementary_Table_1

## Data Availability

This study utilized datasets previously published in two distinct studies [33, 34]. The metagenomic and metatranscriptomic data for the ganjang bacterial community are available under the NCBI BioProject accession number PRJNA613738. Metatranscriptomic data for a synthetic community comprising two bacterial species can be accessed via NCBI BioProjects PRJNA675662. Complete genomes for these bacterial species are available from NCBI BioProjects under the accession numbers PRJNA225 and PRJNA267, respectively. The code for the IMIC approach, along with the scripts used to generate the results presented in this study, as well as all models developed, are openly accessible in the following GitHub repository: https://github.com/YunliEricHsieh/IMIC.
