# Supplementary material for: Integration of metatranscriptomics data improves the predictive capacity of microbial community metabolic models
Source: ISME J. 2025 May 31;19(1):wraf109. doi: 10.1093/ismejo/wraf109 (PMC12203112; doi:10.1093/ismejo/wraf109)
Supplement: Supplementary_Files_wraf109 [file supplementary_files_wraf109.pdf]

Supplementary information

Integration of metatranscriptomics data improves the predictive capacity of microbial community metabolic models

Yunli Eric Hsieh[1,2,3], Kshitij Tandon[3], Heroen Verbruggen[3,4], Zoran Nikoloski[1,2#]

[1] Systems Biology and Mathematical Modeling Group, Max Planck Institute of Molecular Plant Physiology, Potsdam, Germany

[2] Institute of Biochemistry and Biology, Bioinformatics Department, University of Potsdam, Potsdam, Germany

[3] School of BioSciences, The University of Melbourne, Parkville, VIC, Australia

[4] CIBIO, Centro de Investigação em Biodiversidade e Recursos Genéticos, InBIO Laboratório Associado, Campus de Vairão, Universidade do Porto, 4485-661 Vairão, Portugal

#Address correspondence to Zoran Nikoloski, Nikoloski@mpimp-golm.mpg.de

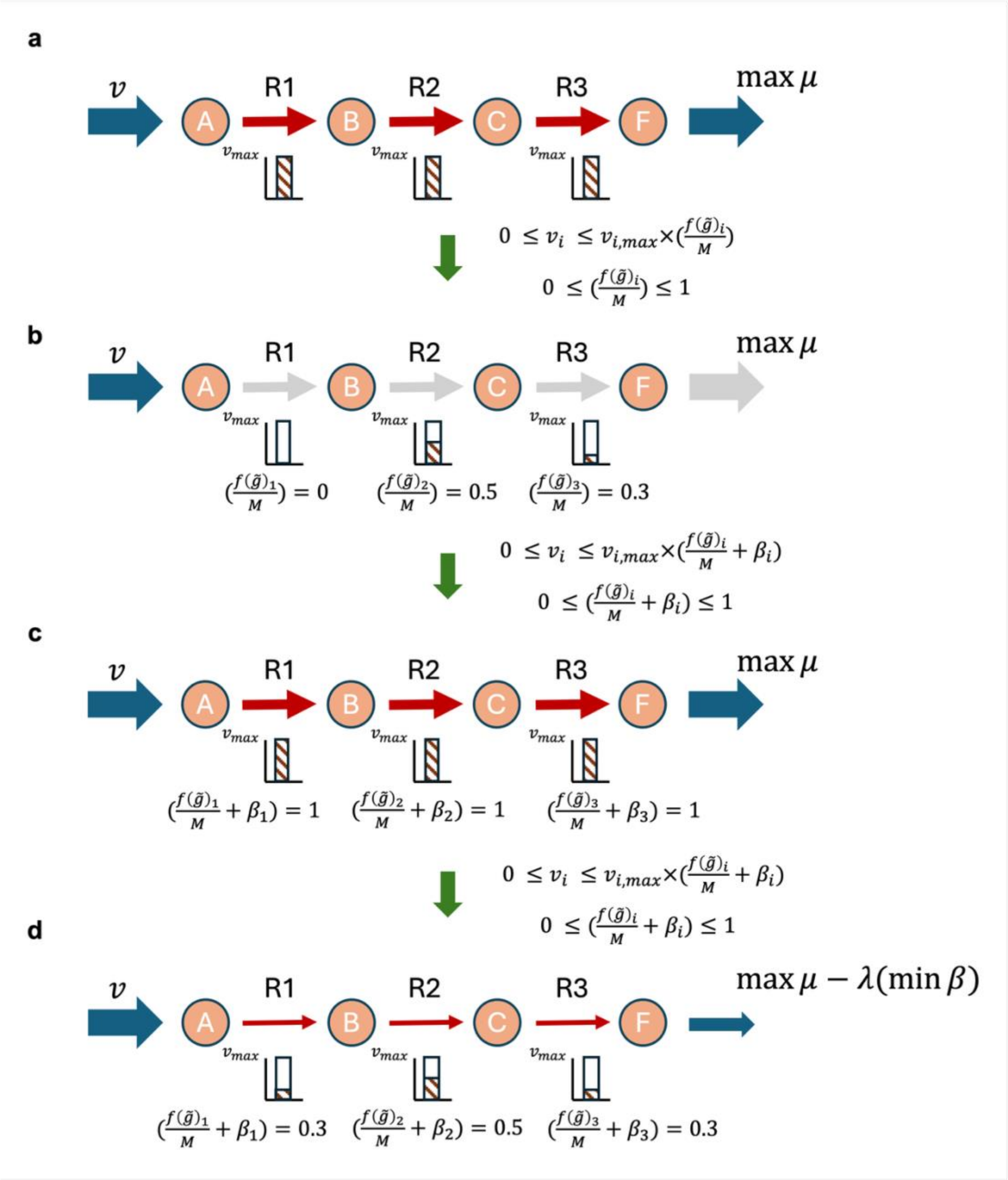

Supplementary Figure 1. Schematic representation of the trade-off space in IMIC. This figure illustrates the relaxation approach employed in IMIC using a simplified reaction network leading to biomass production. R1, R2, and R3 represent upstream reactions contributing to biomass formation. (a) The unconstrained scenario, where reaction fluxes proceed without any restrictions. (b) The application of transcriptomic constraints, where the upper bounds of R1, R2, and R3 are scaled by their respective rescaling factors, $f(\tilde{g})$. In this case, $f(\tilde{g})_1$ is zero, resulting in the complete blockage of R1, which subsequently prevents flux through downstream reactions, including biomass production. (c) To mitigate overly restrictive constraints, a relaxation parameter, β, is

introduced, allowing all reactions to reach their maximum flux potential and restoring biomass production. However, excessive relaxation can lead to overestimation of reaction activity. (d) To achieve a balance between maximizing biomass flux and minimizing the impact of the relaxation parameter, a balancing factor, λ, is incorporated into the objective function, optimizing the trade-off between growth rate and constraint relaxation.

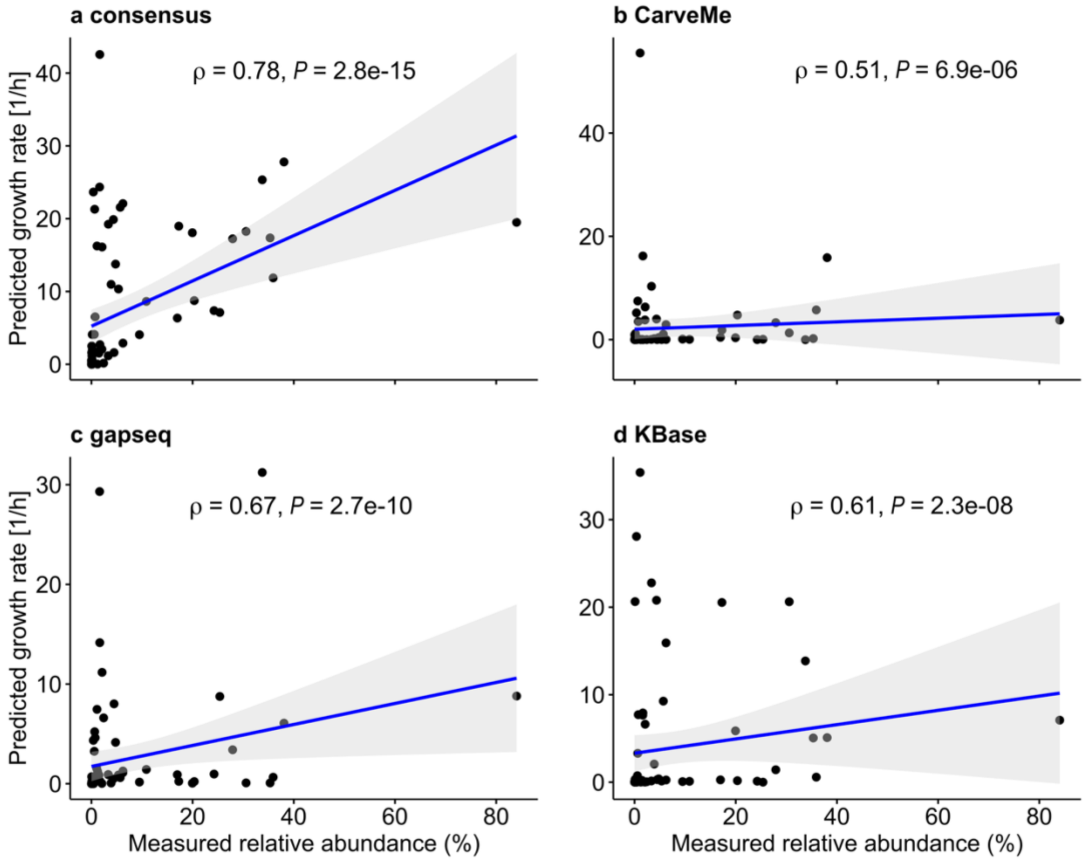

Supplementary Figure 2. Comparative analysis of IMIC performance with models from different reconstruction approaches. The bacterial community models of 14 MAGs were reconstructed from consensus [1], CarveMe [2], gapseq [3], and KBase [4] approaches. The correlation between predicted growth rates from IMIC and measured relative abundance of MAGs was assessed using the Spearman correlation coefficient. IMIC approach was conducted with λ set to 12. Results from the consensus model are shown in Panel (a); CarveMe model results are presented in Panel (b); gapseq model findings are illustrated in Panel (c); and KBase model outcomes are depicted in Panel (d). The shaded area indicates the 95% confidence interval.

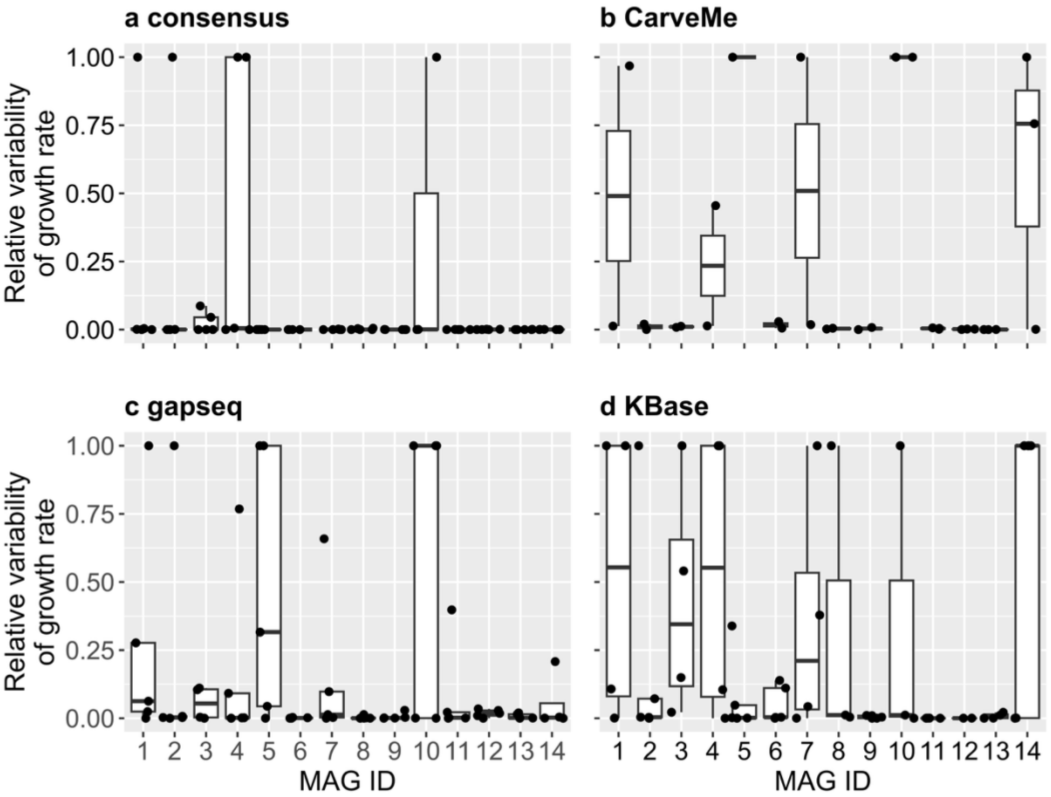

Supplementary Figure 3. Assessment of variability and precision of the predicted growth rates. The relative variability of growth rates for each MAG within the community was quantified as the ratio between the range and the maximum of predicted growth rate, where the range is the difference between the maximum and minimum growth rate. This measure serves as an index of the precision of growth rate predictions. A value closer to zero indicates higher precision in the prediction of growth rates. The analysis was conducted with λ set to 12, which was determined by the sensitivity analysis. Panels (a-d) display the results corresponding to community models generated by consensus [1], CarveMe [2], gapseq [3], and KBase [4] approaches, respectively.

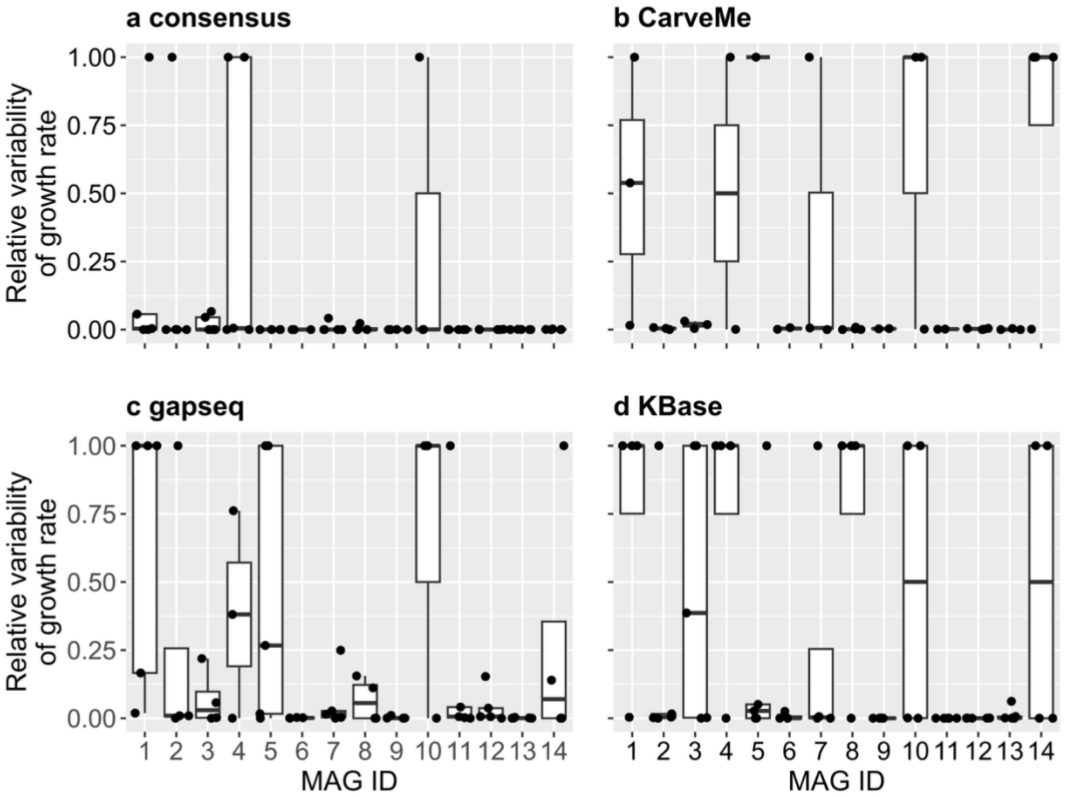

Supplementary Figure 4. Assessment of variability and precision of the predicted growth rates across different lambda values. The balancing factors [12, 20, 15, 25] were applied to the consensus, CarveMe, gapseq, and KBase models to assess the precision of growth rate predictions. These lambda values were selected based on model performance shown in Fig. 3a. Panels (a-d) display the results corresponding to community models generated by consensus [1], CarveMe [2], gapseq [3], and KBase [4] approaches, respectively.

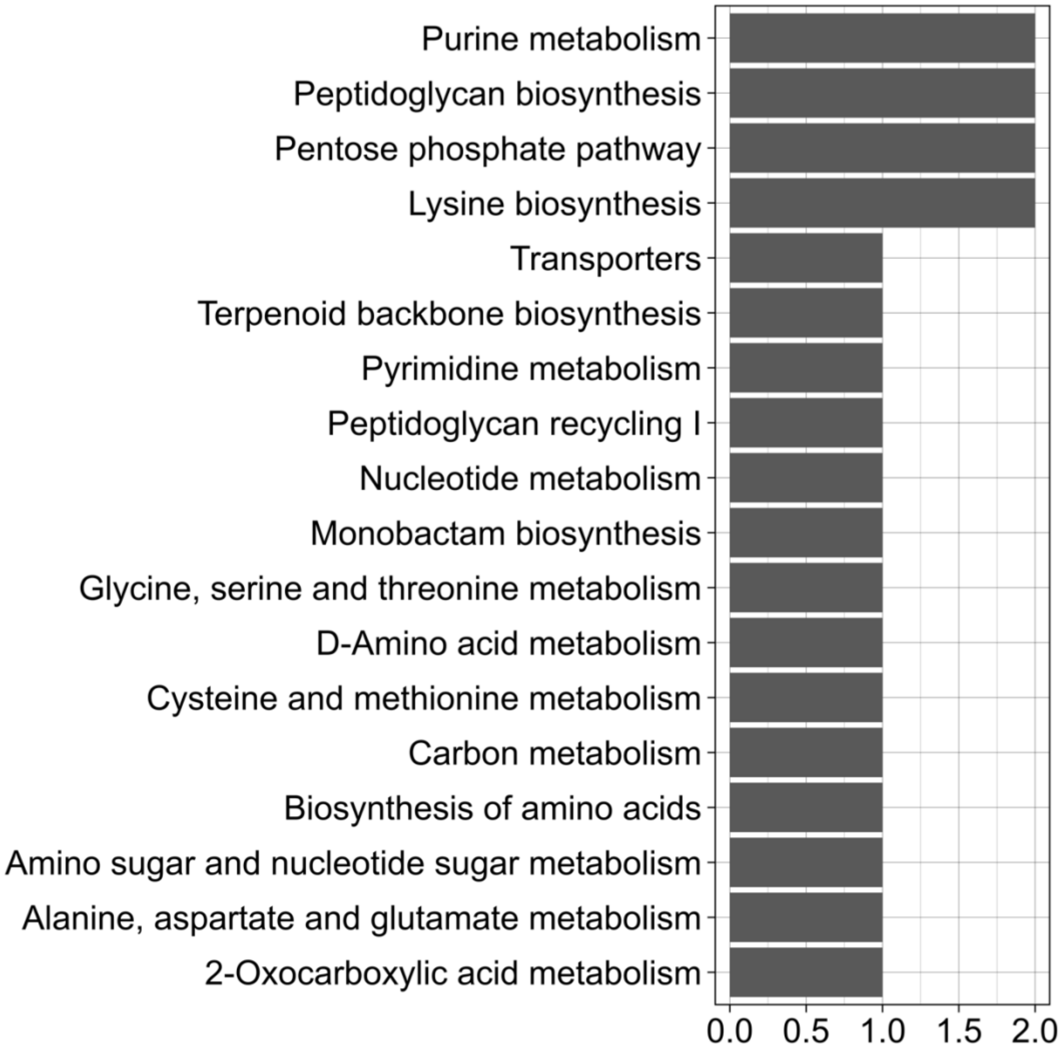

Supplementary Figure 5. The metabolic pathways comprising the 11 key reactions. The metabolic pathways that include the 11 key reactions whose flux values show a high association with the relative abundance of MAGs in the community, were identified using the KEGG database [5-7]. The number presented alongside each pathway denotes the total involvement of these pathways across the identified 11 reactions.

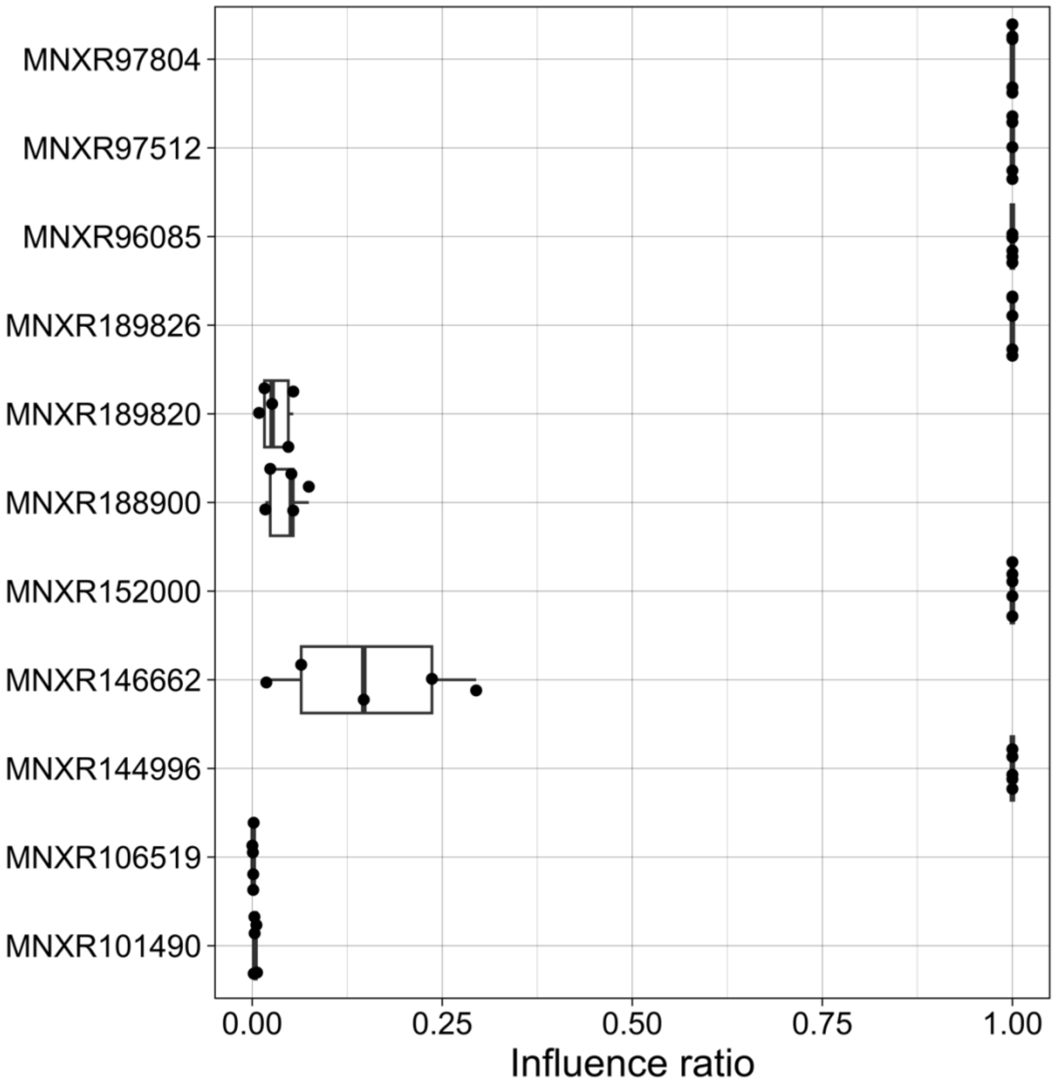

Supplementary Figure 6. Evaluating the impact of the key reactions on community growth rate. This figure quantifies the impact of individually knocking out 11 key reactions, whose flux values have been identified as strongly associated with the relative abundance of MAGs, on community growth rate. To assess this, community growth rates were calculated before and after each reaction was knocked out from the community model. We applied the IMCI with a value of 12 for the balancing factor to determine the difference in the maximum community growth rates with and without reaction knock-out. This difference was then scaled by community growth rate calculated before knocking out the reaction to derive an influence ratio, which varies from 0 to 1. Values closer to 1 indicate a more significant impact on community growth rate.

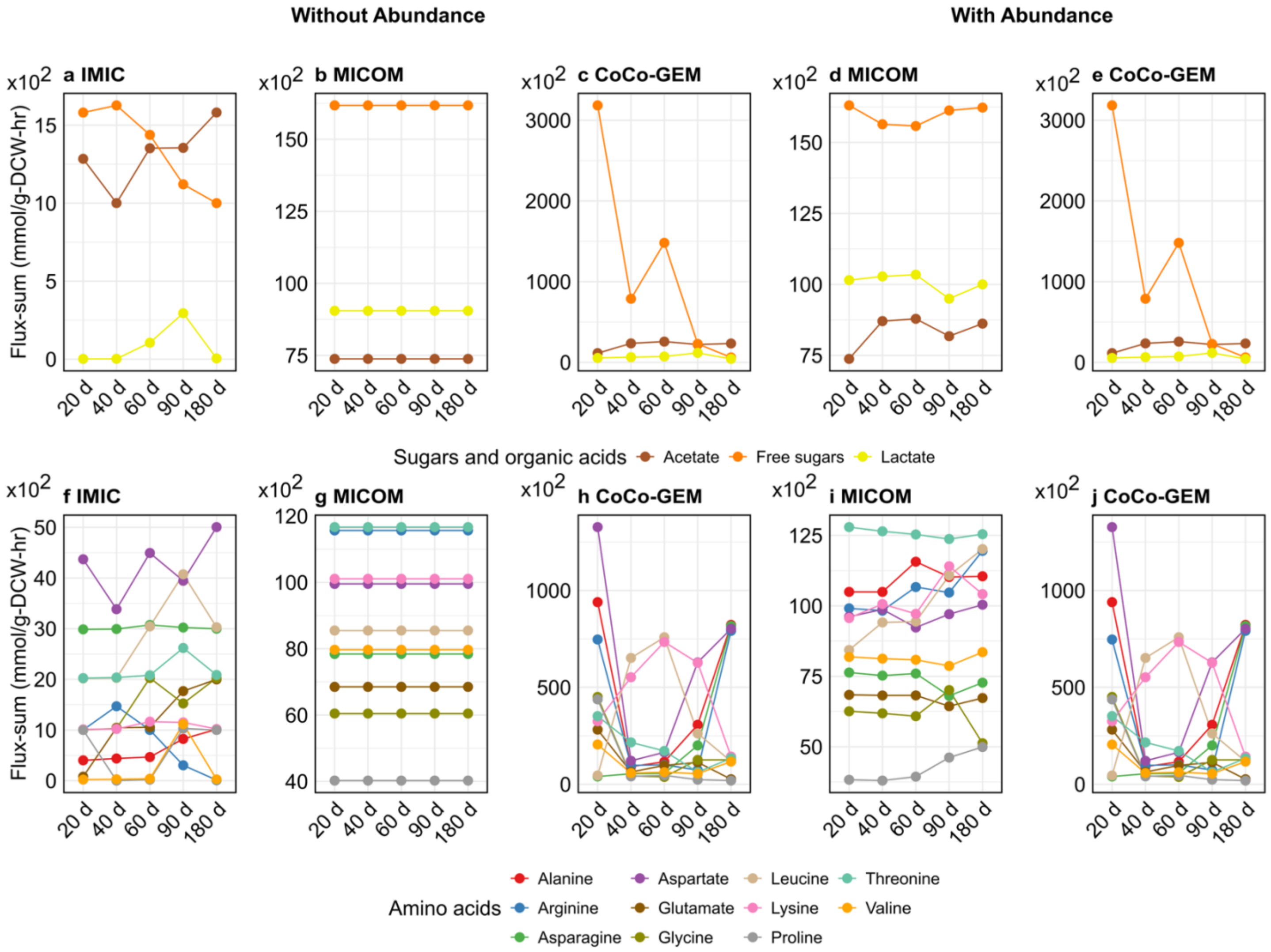

Without Abundance
With Abundance
a IMIC
b MICOM
c CoCo-GEM
d MICOM
e CoCo-GEM
x10^2
Flux-sum (mmol/g-DCW-hr)
20 d
40 d
60 d
90 d
180 d
Sugars and organic acids
Acetate
Free sugars
Lactate
f IMIC
g MICOM
h CoCo-GEM
i MICOM
j CoCo-GEM
Amino acids
Alanine
Arginine
Asparagine
Aspartate
Glutamate
Glycine
Leucine
Lysine
Proline
Threonine
Valine

85

Supplementary Figure 7. Flux sum analysis of free sugars, organic acids, and amino acids. Flux sum values for selected metabolites in the extracellular space of the community model were calculated while solving the optimal objective function of IMIC, MICOM, and CoCo-GEM at each time point. For MICOM and CoCo-GEM, the flux sum changes of the targeted metabolites were analyzed under scenarios with and without the integration of abundance data. The flux sum values of sugars, including mannose, glucose, galactose, and fructose, were aggregated and presented as "free sugars" to illustrate the total flux contribution of these metabolites. Each color in the figure represents a specific metabolite.

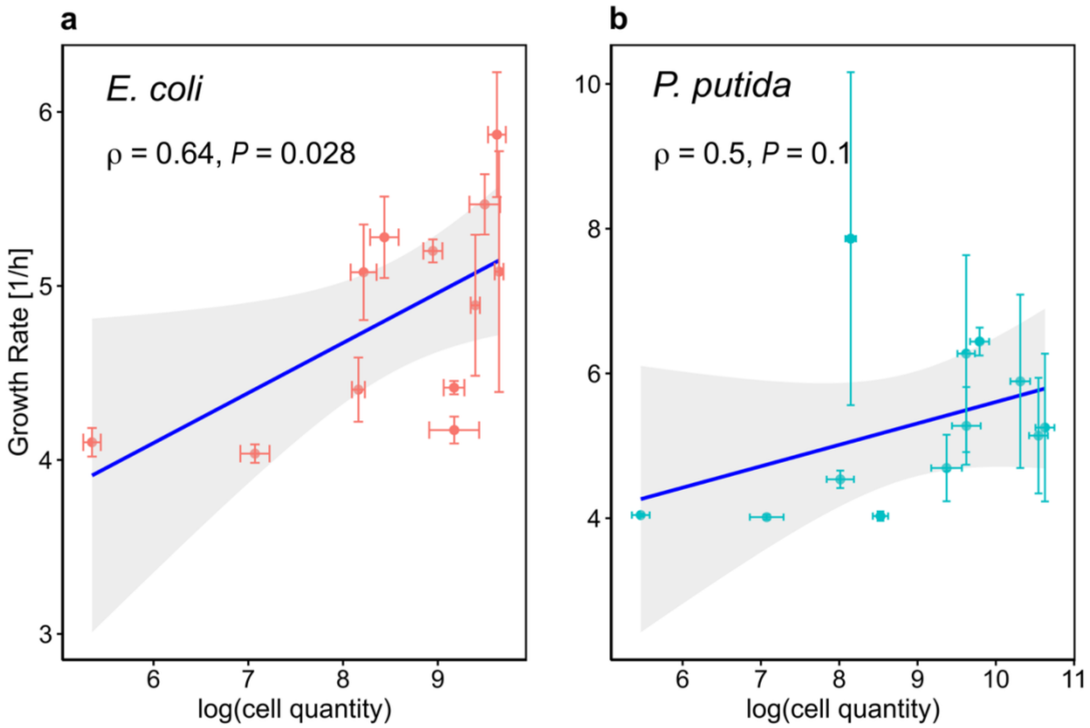

Supplementary Figure 8. Spearman correlation between predicted growth rates and measured cell quantities in *E. coli* and *P. putida*. The association between the predicted growth rates and the log-transformed cell quantities (CFU/mL) was evaluated using the Spearman correlation coefficient. Panel (a) details the correlation outcomes for *E. coli*, and Panel (b) presents the corresponding results for *P. putida*. The shaded area indicates the 95% confidence interval.

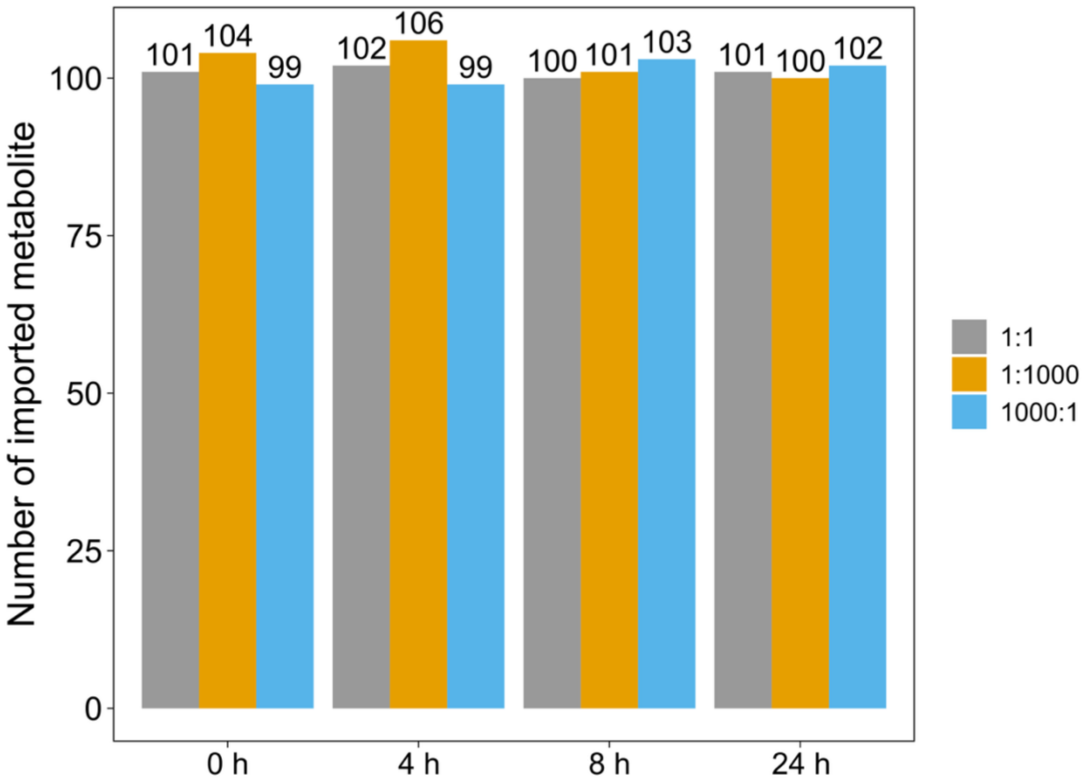

Supplementary Figure 9. Temporal changes in the number of exchanged metabolites for different initial culture ratios. Essential exchanged metabolites were identified by excluding those with minimum flux sum values of zero. The total count of exchanged metabolites within the two-bacterial community model was quantified and compared across different initial culture ratios at the same time point. The three different initial culture ratios (*E. coli* : *P. putida*), 1:1, 1:1000, 1000:1, are shown in different colors, indicated in the legend.

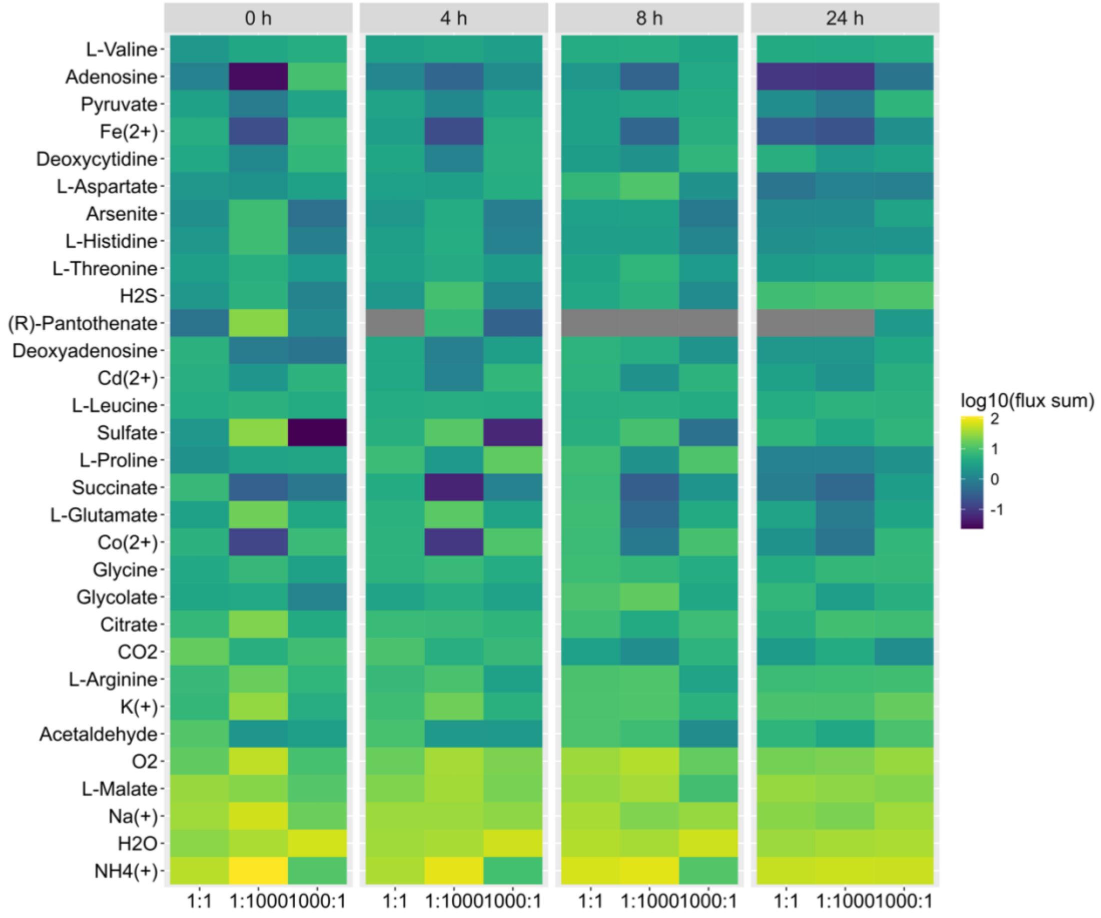

Supplementary Figure 10. Temporal behavior of metabolites exchanged in community model. The top 30 exchanged metabolites were identified by using the minimum flux sum values across three different initial culture ratios of *E. coli* and *P. putida* at four time points. The minimum flux sum values were log-transformed (base 10) for better visualization and comparison.

Supplementary Table 2. Spearman correlation analysis between flux sum values and measured amino acid concentrations. In MICOM, under conditions without the integration of abundance data, the predicted concentrations remained constant across different sampling time points, preventing correlation analysis from being conducted. All *P* values were corrected for multiple hypothesis testing using the Benjamini–Hochberg procedure.

Amino acid	Without Abundance						With Abundance			
	IMIC		MICOM		CoCo-GEM		MICOM		CoCo-GEM	
	rho	*P* value	rho	*P* value	rho	*P* value	rho	*P* value	rho	*P* value
Alanine	0.9	0.2292	-	-	-0.1	1	0.5	0.6188	-0.1	1
Arginine	0.4	0.6315	-	-	-0.6	0.6417	-0.3	0.7517	-0.6	0.6417
Asparagine	0.2	0.8617	-	-	-0.4	0.8119	0.1	0.95	-0.4	0.8119
Aspartate	0.5	0.6188	-	-	0	1	0.5	0.6188	0	1
Glutamate	0.1	0.95	-	-	-0.3	0.9396	-0.5	0.6188	-0.3	0.9396
Glycine	0.7	0.4278	-	-	-0.1	1	-0.4	0.6315	-0.1	1
Leucine	0.9	0.2292	-	-	0.7	0.6417	0.6	0.6188	0.7	0.6417
Lysine	0.8	0.2933	-	-	0.6	0.6417	0.7	0.6188	0.6	0.6417
Proline	-0.5	0.6188	-	-	0.7	0.6417	-1	0.1833	0.7	0.6417
Threonine	-0.9	0.2292	-	-	0.9	0.6417	0.8	0.6188	0.9	0.6417
Valine	0.9	0.2292	-	-	-0.7	0.6417	-0.7	0.6188	-0.7	0.6417

Supplementary Table 3. Pearson correlation analysis between flux sum values and measured amino acid concentrations. In MICOM, under conditions without the integration of abundance data, the predicted concentrations remained constant across different sampling time points, preventing correlation analysis from being conducted. All *P* values were corrected for multiple hypothesis testing using the Benjamini–Hochberg procedure.

Amino acid	Without Abundance						With Abundance			
	IMIC		MICOM		CoCo-GEM		MICOM		CoCo-GEM	
	r	*P* value	*r*	*P* value	*r*	*P* value	*r*	*P* value	*r*	*P* value
Alanine	0.76	0.4058	-	-	-0.36	0.6026	0.64	0.559	-0.36	0.6026
Arginine	0.28	0.648	-	-	-0.96	0.099	-0.21	0.7996	-0.96	0.099
Asparagine	0.38	0.6073	-	-	-0.58	0.5712	0.46	0.6828	-0.58	0.5712
Aspartate	0.36	0.6073	-	-	-0.13	0.8297	0.37	0.7331	-0.13	0.8297
Glutamate	0.57	0.4341	-	-	-0.77	0.4345	-0.32	0.7331	-0.77	0.4345
Glycine	0.70	0.4058	-	-	-0.49	0.6026	-0.16	0.7996	-0.49	0.6026
Leucine	0.73	0.4058	-	-	0.57	0.5712	0.56	0.6044	0.57	0.5712
Lysine	0.66	0.4058	-	-	0.42	0.6026	0.68	0.559	0.42	0.6026
Proline	-0.70	0.4058	-	-	0.37	0.6026	-0.97	0.0563	0.37	0.6026
Threonine	-0.63	0.4066	-	-	0.73	0.4345	0.75	0.559	0.73	0.4345
Valine	0.78	0.4058	-	-	-0.79	0.4345	-0.63	0.559	-0.79	0.4345

Supplementary Table 4. The summary statistic of Spearman correlation and Pearson correlation across all the figures.

Figures	Spearman correlation		Pearson correlation	
	rho	*P* value	*r*	*P* value
Fig. 2a	0.96	< 2.2e-16	0.93	2.2e-16
Fig. 2f	0.78	2.8e-15	0.46	6.5e-05
Fig. 4a	0.29	0.074	0.35	0.029
Fig. 4c	0.0035	0.98	-0.11	0.51
Fig. 4d	0.32	0.046	0.24	0.14
Fig. 4e	0.32	0.044	0.24	0.14
Fig. 4f	-0.024	0.89	0.00091	1
Fig. 4h	-0.14	0.39	-0.24	0.13
Fig. 4i	0.33	0.04	0.3	0.066
Fig. 4j	0.34	0.032	0.19	0.24
Fig. 4k	-0.23	0.17	0.01	0.95
Fig. 4m	-0.17	0.29	-0.19	0.24
Fig. 4n	0.32	0.05	0.14	0.4
Fig. 4o	0.3	0.065	0.087	0.6
Fig. 4p	0.039	0.81	-0.031	0.85
Fig. 4q	-0.048	0.77	0.037	0.82
Fig. 4r	-0.19	0.24	-0.15	0.36
Fig. 4s	0.33	0.038	0.28	0.09
Fig. 4t	0.27	0.096	0.11	0.51
Fig. S2a	0.78	2.8e-15	0.46	6.5e-05
Fig. S2b	0.51	6.9e-06	0.068	0.57
Fig. S2c	0.67	2.7e-10	0.26	0.03
Fig. S2d	0.61	2.3e-08	0.15	0.22
Fig. S8a	0.64	0.028	0.61	0.035
Fig. S8b	0.5	0.1	0.39	0.21

Reference

1. Wendering P, Nikoloski Z. COMMIT: Consideration of metabolite leakage and community composition improves microbial community reconstructions. *PLoS Comput Biol* 2022;18:e1009906. https://doi.org/10.1371/journal.pcbi.1009906
2. Machado D, Andrejev S, Tramontano M, Patil KR. Fast automated reconstruction of genome-scale metabolic models for microbial species and communities. *Nucleic Acids Res* 2018;46:7542-53. https://doi.org/10.1093/nar/gky537
3. Zimmermann J, Kaleta C, Waschina S. gapseq: Informed prediction of bacterial metabolic pathways and reconstruction of accurate metabolic models. *Genome Biol* 2021;22:81. https://doi.org/10.1186/s13059-021-02295-1
4. Arkin AP, Cottingham RW, Henry CS, Harris NL, Stevens RL, Maslov S et al. KBase: The united states department of energy systems biology knowledgebase. *Nat Biotechnol* 2018;36:566-69. https://doi.org/10.1038/nbt.4163
5. Kanehisa M, Goto S. Kegg: Kyoto encyclopedia of genes and genomes. *Nucleic Acids Res* 2000;28:27-30. https://doi.org/10.1093/nar/28.1.27
6. Kanehisa M. Toward understanding the origin and evolution of cellular organisms. *Protein Sci* 2019;28:1947-51. https://doi.org/10.1002/pro.3715
7. Kanehisa M, Furumichi M, Sato Y, Kawashima M, Ishiguro-Watanabe M. Kegg for taxonomy-based analysis of pathways and genomes. *Nucleic Acids Res* 2022;51:D587-D92. https://doi.org/10.1093/nar/gkac963
